# The synergistic effect of chlorotoxin-mApoE in boosting drug-loaded liposomes across the BBB

**DOI:** 10.1186/s12951-019-0546-3

**Published:** 2019-11-11

**Authors:** Beatrice Formicola, Roberta Dal Magro, Carlos V. Montefusco-Pereira, Claus-Michael Lehr, Marcus Koch, Laura Russo, Gianvito Grasso, Marco A. Deriu, Andrea Danani, Sandrine Bourdoulous, Francesca Re

**Affiliations:** 1 School of Medicine and Surgery, University of Milano-Bicocca, Via Raoul Follereau 3, 20854 Vedano al Lambro, MB Italy; 2 Department of Drug Delivery, Helmholtz Institute for Pharmaceutical Research Saarland, Saarland University, 66123 Saarbrücken, Germany; 3 Leibniz Institute for New Materials, Campus D2 2, 66123 Saarbrücken, Germany; 4 Bio Organic Chemistry Laboratory, Department of Biotechnology and Biosciences, University of Milano-Bicocca, Via Raoul Follereau 3, 20854 Vedano al Lambro, MB Italy; 5 Istituto Dalle Molle di Studi Sull’Intelligenza Artificiale (IDSIA), Scuola Universitaria Professionale Della Svizzera Italiana (SUPSI), Università Della Svizzera Italiana (USI), Manno, Switzerland; 6 Department of Mechanical and Aerospace Engineering (DIMEAS), Politecnico di Torino, Corso Duca degli Abruzzi 24, 10128 Turin, Italy; 7 U1016, Institut Cochin, Inserm, Paris, France

**Keywords:** Nanoparticles, Liposomes, Brain, Blood–brain barrier, Drug delivery, Doxorubicin, Chlorotoxin, Glioblastoma

## Abstract

We designed liposomes dually functionalized with ApoE-derived peptide (mApoE) and chlorotoxin (ClTx) to improve their blood–brain barrier (BBB) crossing. Our results demonstrated the synergistic activity of ClTx-mApoE in boosting doxorubicin-loaded liposomes across the BBB, keeping the anti-tumour activity of the drug loaded: mApoE acts promoting cellular uptake, while ClTx promotes exocytosis of liposomes.

## Main text

The blood–brain barrier (BBB) penetration of drugs is one of the biggest challenges in the development of therapeutics for central nervous system (CNS) disorders [1].

The complexity of the BBB hampers the CNS drug delivery: it is formed by specialized brain capillary endothelial cells in direct communication with other cells of the CNS, with the circulating immune cells, and with the peripheral tissues via macromolecules exchange with the blood [2].

However, the BBB targeting and crossing, exploiting different mechanisms, remains the most promising strategy to deliver drugs to the brain without distrupting the barrier [3].

In this context, the incorporation of drugs in nanoparticles allowed the enhancement of drug permeation across the BBB, achieving a more favourable drug pharmacokinetics. Moreover, through the multiple functionalization of nanoparticles surface, a more targeted delivery can be attained [4].

We have reported that liposomes functionalized with a modified Apolipoprotein E-derived peptide (CWG-LRKLRKRLLR; mApoE) are able to cross intact the BBB and to deliver the drug cargo into the brain, using both in vitro and in vivo models [5–8]. However, in animal models, the amount of mApoE-LIP reaching the brain after peripheral administration is 0.2–0.3% of injected dose [7, 8]. This order of magnitude should be not enough to achieve a therapeutic effect of carried drugs for the treatment of CNS diseases. Moreover, it should be pointed out that also the BBB alterations, in aging or disease, could limits the drug delivery [9]. To this purpose, we have reported on the reduction of brain penetration of mApoE-LIP in animal models of Alzheimer’s disease, respect to healthy control mice [10].

In the present work, we aim to improve the performance of mApoE-LIP in BBB crossing by adding the neurotoxin, chlorotoxin (ClTx), as second surface functionalization of LIP. ClTx is a 36-amino acid peptide isolated from the venom of Giant Yellow Israeli scorpion. Since the first isolation, about 25 years ago to date, ClTx has awakened strong interest in oncology due to its high binding affinity to cancer cells, brain tumours included [11–13]. It has been shown that ClTx is able to overcome the BBB in in vivo models without damaging it, but the mechanism by which ClTx crosses the BBB is not fully understood [14, 15].

We designed, synthesized and characterized LIP dually functionalized with mApoE peptide and with a lipid-modified ClTx. Furthermore, we studied the ability of these nanovectors to cross the BBB in vitro, carrying doxorubicin (DOX) payload as a drug model.

**Fig. 1:**
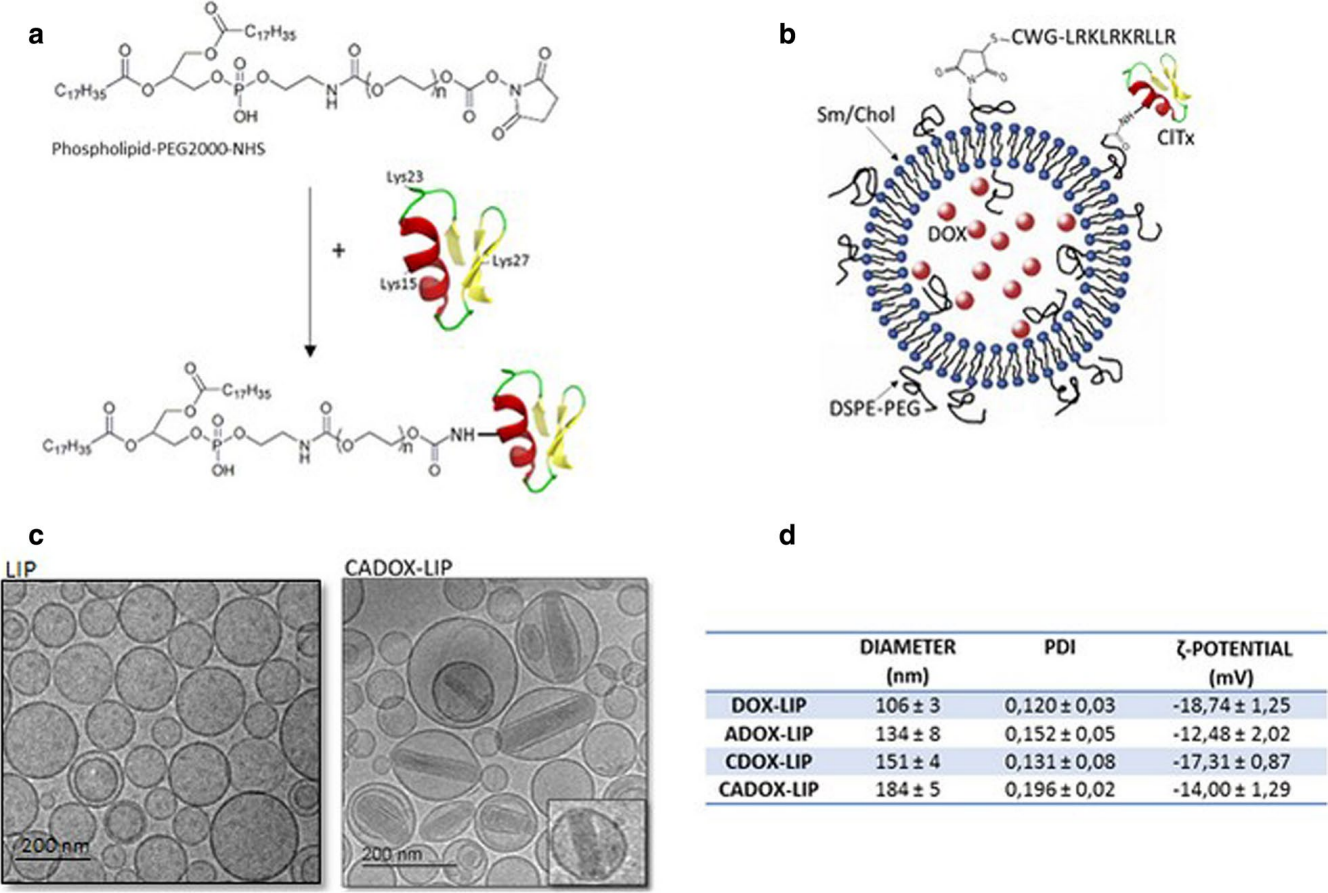
**a** Conjugation reaction of ClTx to DSPE-PEG-NHS. **b** Graphical representation of CADOX-LIP-LIP. **c** Cryo-TEM images of non-functionalized LIP and CADOX-LIP-LIP. Image inset is a magnification of a single liposome carrying DOX. **d** Characterization of LIP by Dynamic Light scattering and *ζ*-potential analyser. Data are expressed as a mean ± SD of at least three independent LIP preparation, each of them in triplicates

The ClTx was chemically modified by attaching 1,2-distearoyl-sn-glycero-3-phosphoethanolamine-polyethylene glycol 2000 with active succinimidyl ester (DSPE-PEG-NHS) via the amide bond formation reaction on Lys15, Lys23 and Lys27 of the peptide (Fig. 1a), following the procedure previously described [16] (Additional file 1).

Liposomes, composed of cholesterol/sphingomyelin/DSPE-PEG-maleimide (48.75/48.75/2.5 molar ratio) [5], were prepared by extrusion procedure through 100-nm filter pores at 60 ± 4 °C (Tc sphingomyelin = 54 °C; Tc DSPE = 74 °C) in 500 mM ammonium sulphate pH 5.5. These non-functionalized liposomes were defined as LIP. LIP were then dialyzed against 10 mM Hepes, 150 mM NaCl pH 7.4 in order to obtain a pH gradient between inner and outer lipid bilayer. DOX was incorporated in LIP core by remote-loading (DOX-LIP) [16]. Subsequently, mApoE peptide with a C-terminal cysteine (Cys) was conjugated to the LIP surface through the maleimide-thiol coupling reaction using an excess of the peptide [5, 6]. These mApoE-LIP embedding DOX have been named as ADOX-LIP. These particles, or DOX-LIP, were functionalized with lipid-ClTx by post-insertion procedure, generating CDOX-LIP (ClTx-DOX-LIP) and CADOX-LIP-LIP (ClTx-mApoE-DOX-LIP) (Fig. 1b). LIP were purified by size-exclusion chromatography and physico-chemically characterized. The yield of DOX encapsulation into LIP was 72 ± 10% and the final preparations contained 210 ± 15 µg of DOX/µmol of lipids. The yield of LIP surface functionalization with mApoE peptide was 63 ± 3%, according to previously published data [5, 6]. The post-insertion yield of lipid-ClTx was 70 ± 6%. Since the post-insertion of a new lipid is expected to alter the membrane of the LIP [17], which may cause leakage of the entrapped drug, the DOX release after functionalization with ClTx was measured. No significant leakage of DOX was observed after lipid-ClTx post-insertion. Cryo-TEM results (Fig. 1c) showed particles with a mean diameter in the order of 200 nm, predominantly unilamellar structure (> 80%). The morphological changes of CADOX-LIP, compared to empty LIP, are due to the encapsulation of the drug, generating the previously reported “coffee-bean” shape [18], with DOX/sulfate co-crystals inside LIP core. All samples displayed a size < 200 nm, were monodispersed (polydispersity index, PDI < 0.2) and negatively charged (Fig. 1d). These parameters remained constant for up to 15 days within the experimental error (< 3.1% of variation) (Additional file 1). Considering that, ~ 100,000 lipids are in the outer layer of a 200-nm diameter LIP, which contain 2.5 mol  % of DSPE-PEG-maleimide, the mApoE density after incubation is of ~ 1200 peptide molecules per single LIP. The ClTx density ranging from 200–300 peptide molecules per single LIP.

**Fig. 2:**
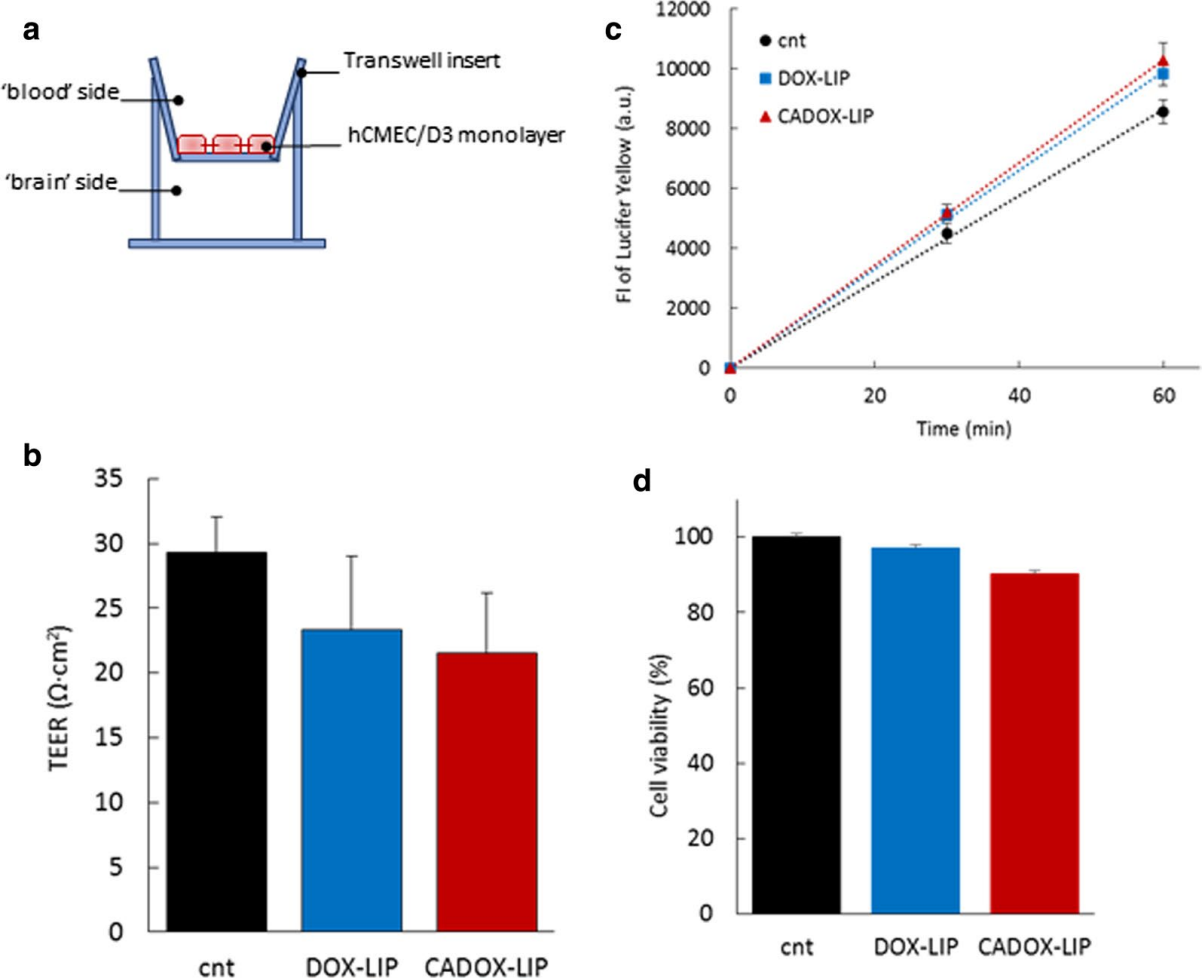
**a** Graphical representation of the transwell system used to mimic the BBB. **b** TEER values. **c** Fluorescence intensity of LY in the ‘brain’ side of the transwell system before and after 1 h of incubation with DOX-LIP or CADOX-LIP. **d** hCMEC/D3 cell viability assessed by MTT assay after 24 h of incubation with DOX-LIP or CADOX-LIP. Data are expressed as a mean ± SD of at least three independent LIP preparation, each of them in triplicates

The capacity of CADOX-LIP to translocate across brain endothelial cells, carrying drug payload, was assessed in an in vitro human BBB model. Immortalized human brain capillary endothelial cells (hCMEC/D3) were cultured on semipermeable membrane filters of a transwell system (Fig. 2a) (Additional file 1). First, the effect of liposomes treatment on hCMEC/D3 features was assessed by measuring: (i) transendothelial electrical resistance (TEER); (ii) endothelial permeability (EP) to the paracellular probe lucifer yellow (LY); (iii) cell viability by MTT assay; (iv) morphological cell features by optical microscopy [7, 19] (Additional file 1). Free DOX showed an important toxic effect on BBB model, as reflected by alterations of bioelectrical (TEER = 16 ± 3 Ω cm^2^, compared to 29 ± 3 Ω cm^2^ of untreated cells), functional (EP to LY = 3.06 ± 0.2·10^−3^ cm/min, respect to 1.87 ± 0.6·10^−3^ cm/min of untreated cells) and structural properties (< 20% of viable cells) of hCMEC/D3 monolayer. These effects were prevented by incorporation of DOX in the LIP core, indicating that the BBB integrity can be preserved. Moreover, the surface functionalization of LIP with mApoE and ClTx did not shown significant toxic effect on the BBB model features (Fig. 2b–d).

**Fig. 3:**
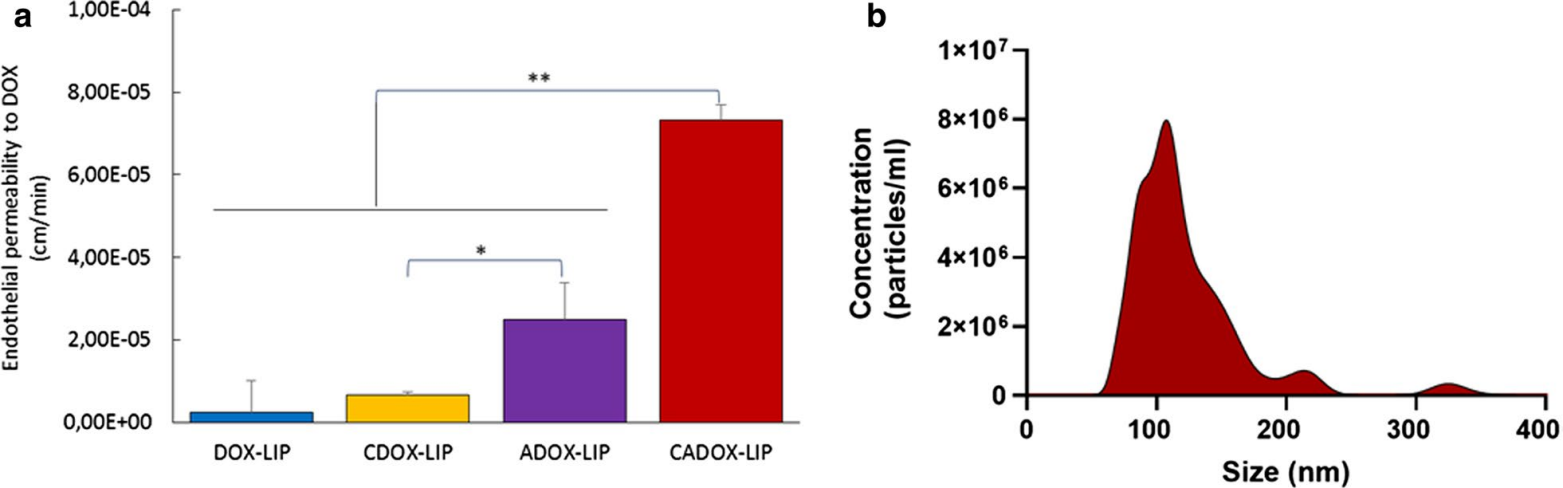
Permeability of LIP across human in vitro BBB cellular model. **a** Endothelial permeability to DOX in different LIP formulations. **b** Analysis of CADOX-LIP-LIP size and quantity in the basolateral compartment of the transwell system, after BBB crossing, measured by nanosight. Data are expressed as a mean ± SD of at least five independent LIP preparation, each of them in triplicates

Afterwards, the EP to the different LIP formulations was calculated by measuring over time the amount of DOX in the apical (‘blood’ side) and in the basolateral (‘brain’ side) compartment of the transwell system, as reported [20] (Additional file 1). Non-functionalized LIP were not able to transport DOX across the BBB model in a significant amount, while ADOX-LIP allowed a ten-fold increase of EP to DOX, as expected and already reported using a different drug [6]. Unexpectedly, CDOX-LIP did not shown a substantial enhancement of DOX passage across the cells monolayer, suggesting that the capability of ClTx to permeate the BBB is too weak. This is in agreement with previously published data, showing that ClTx is able to cross human endothelial cells by active transcytosis, but this passage is probably not enough to deliver drugs across the BBB [21]. Interestingly, CADOX-LIP were able to boost (~ 30-fold increase) DOX passage across the BBB, respect to DOX-LIP. Moreover, the increase of DOX passage was 3-times and 10-times higher when incorporated in CADOX-LIP, compared to ADOX-LIP and CDOX-LIP, respectively (Fig. 3a). This suggests the existence of a synergistic effect between the two ligands: ClTx and mApoE. Nanosight analysis of LIP passed through the cells monolayers (i.e. in the basolateral compartment of the transwell system) suggested that LIP remain intact after BBB crossing (Fig. 3b), thus excluding the possibility to have follow only the passage of DOX released from LIP. The EP to free DOX was 1.27 ± 0.2 × 10^−3^ cm/min, a lot greater than when it was incorporated in LIP. This is almost certainly due to its toxic effect on hCMEC/D3 monolayer, as described above. It is important to point out that these results were obtained using endothelial cells grown in a monoculture that does not properly represent the in vivo BBB tightness. Unfortunately, we could not performed these experiments in advanced multicellular BBB models because of the liposomes uptake by astrocytes, which in turn affects the calculation of EP.

**Fig. 4:**
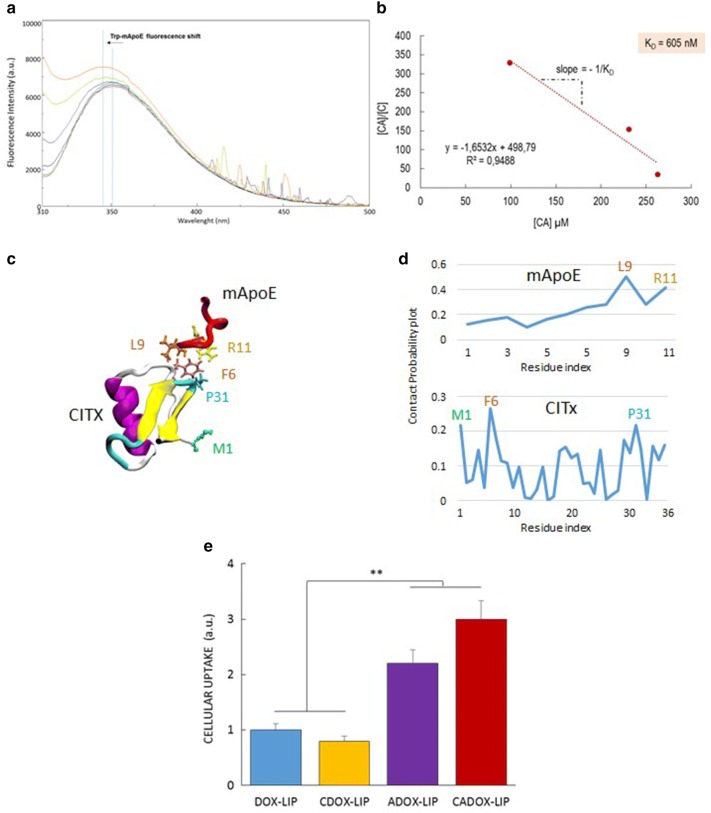
**a** Trp fluorescence of 30 μM mApoE after incubation with different doses of ClTx ranging from 0 to 30 μM. **b** Scatchard plot to determine the binding constant between mApoE e ClTx in solution, where [CA] is the concentration of ClTx-mApoE complex and [C] is concentration of ClTx. **c** Visual Inspection of mApoE-CITX interaction by MDS. Residues mainly responsible for the protein–protein interaction are also highlighted. **d** Contact probability plot reporting the probability of each protein residue to be part of the protein–protein contact surface. **e** Cellular uptake of DOX in different LIP formulations. Data are expressed as a mean ± SD of triplicates

In order to investigate the ClTx-mApoE synergy in enhancing the LIP passage across the BBB, we evaluated the mApoE-ClTx binding by spectrofluorimetry, the type of interaction by computational docking and molecular modelling analysis and the cellular uptake of DOX (Additional file 1). Following the tryptophan (Trp) fluorescence shift of mApoE after binding with increasing amounts of ClTx, we observed that mApoE-ClTx are able to interact between them, with a calculated affinity constant of K_a_ = 1.65 ± 0.3x10^−3^ nM (Fig. 4a, b). Molecular dynamics simulations (MDS) confirmed that the two proteins are able to interact (Fig. 4c) and the residues mainly involved in the interaction are M1, F6, P31 of ClTx and L9, R11 of mApoE (Fig. 4d), which are not engaged in reactions for LIP functionalization. However, the interaction is fleeting and not very stable. In fact, it is possible to observe many protein–protein breakdown events in the movie (Additional file 2: Movie S1). This is probably due to the strong electrostatic repulsion between the two ligands, since they are both positively charged sequences.

Finally, comparing the cellular internalization of DOX, its uptake was ~ 30% higher when incorporated in ADOX or CADOX-LIP (i.e. 7.80 ± 1.29% and 10.64 ± 0.74% of administered DOX was measured inside the cells, respectively), respect to DOX-LIP and CDOX-LIP (Fig. 4e). The increased DOX uptake in ADOX-LIP and CADOX-LIP supports the higher BBB permeability of these two formulations. Most interestingly, considering that the extent of DOX uptake between ADOX-LIP and CADOX-LIP is similar, and the functionalization of LIP only with ClTx (CDOX-LIP) did not improve the DOX uptake, we can speculate that ClTx is not involved in the cellular internalization. Rather it is possible to assert that ClTx is involved in the LIP egress from the basolateral side of endothelial cells.

Taken together these results suggest that there is not a cooperative action of ClTx and mApoE in entering the endothelial cells, but there is a synergistic activity in BBB crossing. In particular, mApoE acts promoting the interaction of CADOX-LIP with the apical side of human endothelial cells, probably via LDL-receptor mediated endocytosis as already demonstrated [5–7], whereas ClTx acts by boosting CADOX-LIP outside of the endothelial cells, promoting their exocytosis though the basolateral side of cell monolayers.

If we consider that the amount of DOX internalized in hCMEC/D3 is similar between ADOX and CADOX-LIP, we can propose differences in the exocytosis process. In this case, a more effective exocytosis would explain the enhanced transcytosis properties of CADOX-LIP, when compared to CDOX-LIP and ADOX-LIP.

The mechanism by which ClTx is able to promote exocytosis of CADOX-LIP will deserves further investigation. However, data from literature suggest an interaction of ClTx with Annexin A2, which is expressed in vascular endothelial cells, BBB included [22, 23]. Recently, it has been shown that Annexin A2 is involved in the BBB transcytosis processes of CNS pathogens, but not in their cellular adhesion and uptake, suggesting a role of Annexin A2 in the exocytosis pathway across the BBB [24, 25]. Then, ClTx may promote the LIP exocytosis interacting with Annexin A2. This mechanism of action is speculative and the demonstration needs further research to deep this issue, for example by testing alternatives to CITx known for their interaction with Annexin A2.

**Fig. 5:**
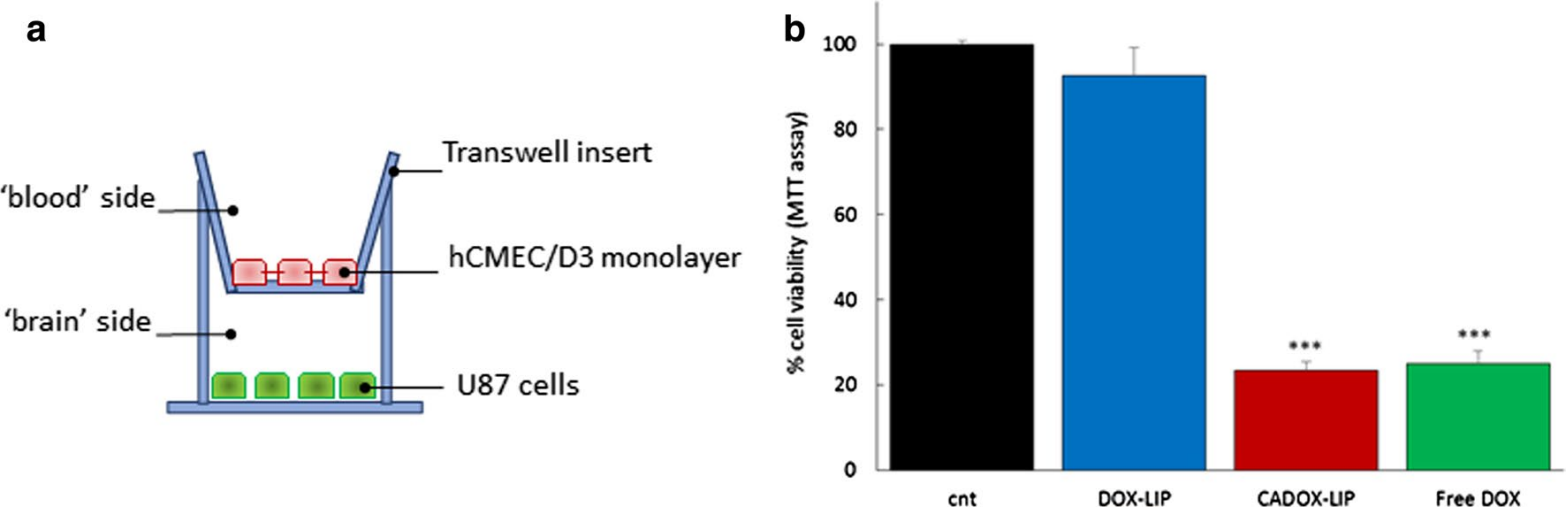
**a** Graphical representation of co-culture transwell model utilized to test the efficacy of LIP after BBB crossing on human glioblastoma U87 cells. **b** Cell viability assessed by MTT assay on U87 cells seeded in the basolateral compartment of the transwell system. Data are expressed as a mean ± SD of triplicates and analysed with unpaired one-tailed Student’s t test. ***p < 0.001

Finally, we tested if the drug embedded in CADOX-LIP was able to retain its ability to target and to suppress the growth of a cellular model of brain tumour. To this purpose, a co-culture transwell system with hCMEC/D3 cells seeded on filter and human U87 glioblastoma cells seeded in the basolateral compartment (Fig. 5a) was prepared. The results (Fig. 5b) showed that after BBB crossing, CADOX-LIP were able to reduce (− 76.6%) the viability of U87 cells seeded in the basolateral compartment, as well as free DOX, but without damaging the endothelial monolayer.

This proofs the capability of CADOX-LIP to carry active DOX to cancer cells in vitro.

In conclusion, our results reveal the synergistic effect of ClTx-mApoE in improving the permeability of drug-loaded LIP across a human cell-based BBB model. These data suggest that the already reported ability of mApoE to cross the BBB can be improved by ClTx, which enhances the exocytosis from hCMEC/D3 cell monolayers. On the other side, mApoE can improve the already reported ability of ClTx to translocate across the BBB in vitro, enhancing its penetration in endothelial cells. All these data support the use of mApoE-ClTx as a dual-targeting ligands to functionalize particles to facilitate the brain delivery of different drug cargoes across the BBB.

## Supplementary information

**Additional file 1.** Materials and methods.

**Additional file 2**. Movie of MDS of mApoE-ClTx interaction.

## Data Availability

All data generated or analysed during this study are included in this published article and its supplementary information files.

## Abbreviations

BBB: blood–brain barrier
CNS: central nervous system
mApoE: modified apolipoprotein E-derived peptide
Cys: cysteine
ClTx: chlorotoxin
LIP: liposomes
DOX: doxorubicin
Lys: lysine
DSPE-PEG: 1,2-distearoyl-sn-glycero-3-phosphoethanolamine-N-[(polyethylene glycol)-2000]
DOX-LIP: liposomes embedding doxorubicin
ADOX-LIP: liposomes functionalized with mApoE and containing doxorubicin
CDOX-LIP: liposomes functionalized with chlorotoxin and containing doxorubicin
CADOX-LIP: liposomes bi-functionalized with chlorotoxin and mApoE, and containing doxorubicin
TEM: transmission electron microscope
hCMEC/D3: human cerebral microvascular endothelial cells/human brain capillary endothelial cells
TEER: transendothelial electrical resistance
EP: endothelial permeability
LY: lucifer yellow
PDI: polydispersity index
MTT: 3-(4,5-dimethylthiazol-2-yl)-2,5-diphenyltetrazolium bromide
MDS: molecular dynamics simulations
Trp: tryptophan
