# Supplementary material for: The synergistic effect of chlorotoxin-mApoE in boosting drug-loaded liposomes across the BBB
Source: J Nanobiotechnology. 2019 Nov 11;17:115. doi: 10.1186/s12951-019-0546-3 (PMC6844026; doi:10.1186/s12951-019-0546-3)
Supplement: Supplementary file 1 — Additional file 1. Materials and methods. [file 12951_2019_546_MOESM1_ESM.docx]

**Supporting Information for:**

**The synergistic effect of chlorotoxin-mApoE in boosting drug-loaded liposomes across the BBB**

**Beatrice Formicola, Roberta Dal Magro, Carlos V Montefusco-Pereira, Claus-Michael Lehr, Marcus Koch, Laura Russo, Gianvito Grasso, Marco Deriu, Andrea Danani, Sandrine Bourdoulous, and Francesca Re**

**Materials**

Cholesterol (Chol), Doxorubicin (DOX), Ultra-low Range Molecular Weight Marker (M.W. 1.060-26.600), EZBlue Gel Staining Reagent and Thiazolyl Blue Tetrazolium Bromide, 4-(2-Hydroxyethyl)piperazine-1-ethanesulfonic acid (Hepes), Triton X-100 were purchased from Sigma-Aldrich (Milano, Italy). 1,2-Distearoyl-sn-glycero-3-phospho-ethanolamine-N[maleimide(polyethyleneglycol)-2000](mal-PEG-DSPE) and sphingomyelin from bovine brain (Sm) were purchased from Avanti Polar Lipids, Inc (Alabaster, AL, USA). 1,2-Distearoyl-sn-glycero-3-phospho-ethanolamine-N [(polyethyleneglycol)-2000] n-hydroxysuccinimide ester (NHS-PEG-DSPE) was purchased from Nanocs (Boston, USA) .Ultrapure and deionized water were obtained from Direct-Q5 system (Millipore, Italy). Transwell permeable supports 0.4 μm polyester membrane 12 mm insert, 12 well plates were from Corning (NY, USA). mApoE peptide (CWGLRKLRKRLLR, MW 1698.18 g/mol) and chlorotoxin (ClTx, MW 4004 g/mol) were synthetized by Karebay Biochem (Monmouth Junction, NJ, USA). Dialysis membranes (cut-off 12000-14000 Da) were purchased from Medicell International Ltd, (London, UK). Penicillin-streptomycin (P/S) solution 100X was purchased from Euroclone (Milan, Italy); PBS 1X, collagen, tripsin/EDTA solution and NuPAGE Bis-Tris (4-12%) were from Invitrogen. The lactate dehydrogenase (LDH) cytotoxicity detection kit was purchased from Roche Diagnostics GmbH (Germany). All other chemicals were of analytical grade and were obtained from either Sigma–Aldrich or Merck.

**Synthesis of DSPE-PEG-ClTx**

The ClTx was conjugated to terminal NHS activated DSPE-PEG_2000_ following the procedure previously,^1^ with small modifications. 0.1 µmol DSPE-PEG-NHS dissolved in CHCl_3_/MeOH (2:1, vol/vol) was dried under N_2_ to remove organic solvents. Then 5 eq (0.5 µmol) of ClTx dissolved in 10 mM Hepes, 150 mM NaCl pH 7.4, was added to the dried lipid. The reaction was conducted under gentle stirring for 90 min at room temperature. The resulting solution was dialyzed against deionized MilliQ water for 48 h in a dialysis tube with cut off molecular weight (MW) of 3500 Da to remove unreacted ClTx. Then the solution was lyophilized and stored at − 20 °C. The DSPE-PEG-ClTx recovered from dialysis tube was quantified by bicinconinic acid assay.

**Preparation of liposomal formulations**

Liposomes (LIP), composed of Sm/CHOL (1:1, mol/mol) added with 2.5 mol% mal-PEG-DSPE, were prepared in ammonium sulphate (500 mM, pH 5.5) by extrusion procedure through polycarbonate membranes of 100 nm diameter pores, under N_2_.^2^ LIP were then dialyzed against 10 mM Hepes, 150 mM NaCl pH 7.4, overnight, and then incubated with DOX (2.7 µmol DOX/ 10 µmol total lipids) for 1 h at 65°C, alternating heating and ultrasounds, to allow the incorporation of DOX in LIP core.^1^ This sample will be referred as DOX-LIP. mApoE peptide was covalently attached on DOX-LIP surface by thiol-maleimide coupling between mal-PEG-DSPE and the terminal thiol group of mApoE. This sample will be referred as ADOX-LIP.^3,4^

DSPE-PEG-ClTx was added to ADOX-LIP or to DOX-LIP by post-insertion technique.^5^ The reaction was conducted for 16 h at 41 °C. The resulting preparation was purified by size-exclusion chromatography using Sepharose G-25 fine column (25x1 cm) and 10 mM Hepes, 150 mM NaCl pH 7.4 as eluent, to remove free DOX and unbounded peptides.^2-4^ The final formulations were namely CADOX-LIP and CDOX-LIP.

**Characterization of liposomal formulations**

After LIP purification, the recovered amount of each compound was determined by different techniques. Briefly, DOX loading was quantified spectrofluorometrically (λex = 495 nm; λem =592 nm) after vesicle disruption with 0.1% Triton X-100. The DOX encapsulation yield in each LIP formulation was calculated by comparing fluorescence intensities with a calibration curve for free DOX in 10mM Hepes, 150 mM NaCl pH 7.4. Total phospholipids content was quantified by Stewart Assay.^6^ The attachment of mApoE peptide on LIP surface was monitored by measuring the Trp fluorescence.^2,3^ The bi-functionalization of LIP with mApoE and ClTx was determined by SDS-PAGE gel electrophoresis on precast NuPAGE 4-12% Bis-Tris Gel. Protein bands were visualized by EZ-Blue staining.

Size, and polydispersity index (PI) of LIP formulations were analysed by dynamic light scattering (DLS) technique (Brookhaven Instruments Corporation, Holtsville, NY, USA). ζ-potential was determined by using an interferometic doppler velocimetry with the same instrument equipped with ZetaPALS device. The stability of LIP dissolved in 10 mM Hepes, 150 mM NaCl pH 7.4 was determined by following size and PDI by DLS for at least 1 week. ^2-4^

Size and morphology of LIP formulations was also characterized by cryo-TEM analysis. Briefly, 3 µL of 5 mM LIP was employed onto holey carbon film (type S147-4 from Plano GmbH, Wetzlar, Germany), plotted for 2 s into a thin film and plunged into liquid ethane using a cryo plunge 3 system from Gatan (Pleasanton, CA, USA) operating at T=108 K. Imaging was performed by transferring the cryo-samples in a cryo-TEM sample holder (Gatan model 914) and then by using bright-field imaging low-dose mode (JEOL JEM-2100 LaB6) at T=100 K and 200 kV accelerating voltage. On the recognition of encapsulated drug, an increasing electron dose was applied to the cryo-samples and imaged. The range used was from 30 to 300 pA/cm^2^, as indicated by the JEOL user interface.

**Cell lines and culturing conditions**

Human cerebral microvascular endothelial (hCMEC/D3) cells were obtained from Institut National de la Santé et de la Recherche Médical ([INSERM] Paris, France). Cells at passages between 25 and 35 were grown on tissue culture flask, covered with 0.1 mg/ml rat tail collagen type I. hCMEC/D3 were maintained in Endothelial Basal Medium (EBM-2) supplemented with 5% FBS-PAA, 1% chemically defined lipid concentrate (CDLC), 1% P/S, 10 mM Hepes 10 mM, 5 µg/ml ascorbic acid 5 µg/ml, 1 ng/ml basal fibroblast growth factor (bFGF) 1 ng/ml, 1.4 µM hydrocortisone.^4,7^

U87-MG glioblastoma cells were purchased from da American Type Culture Collection (ATCC, VA, USA) and were grown in DMEM High Glucose, 10% FBS, 1% P/S, 1% glutamine.^8^

**In vitro BBB models**

The *in vitro* BBB model was prepared using a Transwell^TM^ system. hCMEC/D3 were cultured on inserts pre-coated with rat tail collagen type I solution (150 µg/ml) at a density of 5.6 x 10^4^ cells/cm^2^ on the apical side of the semi-permeable filter for 7 days according to the literature.^4,7^ Cells culture medium was changed every two days. hCMEC/D3 monolayers formation was monitored by measuring the transendothelial electrical resistance (TEER) by EVOMX meter, STX2 electrode (World Precision Instruments, Sarasota, FL, USA) as previously described.^4^ At day 7 of culture the endothelial permeability (EP) of the paracellular tracer Lucifer Yellow (LY) was evaluated by measuring LY fluorescence in the basolateral compartment as previously reported.^7,9^

The co-culture was prepared by seeding 5.6x 10^4^ cells/cm^2^ hCMEC/D3 on the apical side of the semi-permeable filter of the Transwell^TM^ system pre-coated with rat tail collagen type I solution (150 µg/ml). After 48 h, 4.6 x 10^4^ cells/cm^2^ U87-MG cells were seeded in the bottom chamber of the Transwell^TM^ system. Cells were co-cultured in their own culture medium for additional 5 days at 37 °C, 5% CO_2_. Cells culture media were changed in day 2, 4 and 7.

**Impact of free DOX and LIP formulations on cell monolayer properties**

hCMEC/D3 cells were cultured on a transwell system as described above. Bioelectrical, morphological and functional properties of the cell monolayers were checked after 24 h of incubation with free DOX, DOX-LIP or CADOX-LIP in the apical compartment (25 µg/ml of DOX). TEER and EP of LY were determined as described above. Morphological features of cell monolayer were evaluated by examining cells under microscope. Cell viability was also assessed by MTT assay, as described.^4^ Untreated cells were used as a control.

**Liposomes cellular uptake and exchange across the BBB model**

25 µg/ml of DOX, free or incorporated in the different LIP formulations, was added to the apical compartment of transwell system and its fluorescence in the basolateral (‘brain’ side) compartment was measured over time, up to 3 h. EP was calculated as described.^10^ To determine the cellular uptake, at the final time point (3 h) hCMEC/D3 cells were washed with PBS, detached from the transwell inserts with trypsin/EDTA for 15 min at 37 °C and the cell-associated DOX fluorescence was measured, as described above. The integrity of LIP after BBB crossing was evaluated by measuring the LIP size in the basolateral compartment of the Transwell system by Nanoparticle Tracking Analysis (NanoSight NS300, Malvern Panalytical, Malvern, U.K.).

**mApoE-ClTx binding**

The binding of mApoE to ClTx was monitored by Trp fluorescence at 25 °C as described. A stock solution of ClTx (0–30 μM final concentration) was titrated into a 30 μM solution of mApoE.^2-4^ The buffer was 10 mM Hepes, 150 mM NaCl pH 7.4. To ensure equilibration of the mixture, the sample was stirred for 2 min and incubated for 10 min at 25 °C before each measurement. Data were obtained from three independent LIP preparations, each of them in triplicates. One-site biding equation was used to deduce the dissociation constant. The experimental binding curves were fitted by the following equation using a nonlinear regression routine, as described.^11^ Scatchard plot was used to determine the binding constant between mApoE e ClTx in solution, where [CA] is the concentration of ClTx-mApoE complex and [C] is concentration of ClTx.

**Molecular dynamics simulations**

The molecular structures of mApoE (LRKLRKRLLR) and CITX were taken from the Protein Data Bank (PDB ID 2L7B and 6ATW, respectively). The molecular system was solvated in a 8 nm cubic box and neutralized by counterions. Each system consisted of about 50.000 particles. The CHARMM36 force field^12^ was used to define protein topologies and the TIP3P^13^ model was used for the explicit solvent. GROMACS 5.1.4^14^ version was employed for MD simulations. Steepest descent algorithm^15^ was utilized to minimize the system. Then, a 50 ps simulation in NVT ensemble was conducted, by applying the V-rescale algorithm^16^ to keep temperature at 300 K with τ 0.1 ps. In order to increase the statistics, three replicas were generated. A 50 ps simulation in a NPT ensemble for each replica was carried out. Weak coupling algorithms^16,17^ were used for pressure and temperature control. Finally, for each replica 500 ns long MD were simulated coupling the system by Nose-Hoover^18^ and Parrinello-Rahman^17^, for temperature and pressure respectively. The LINCS algorithm^19^ was used to constrain the length of h-bonds. Electrostatics was treated by PME algorithm^20^. Trajectories were extracted every 50 ps of simulation and the Visual Molecular Dynamics (VMD) package^21^ was employed to provide the visual inspection of the simulated systems.

**REFERENCES**

1 Y. Xiang, L. Liang, X. Wang, J. Wang, X. Zhang and Q. Zhang, *Journal of Controlled Release*, 2011, **3**,402-410.

2 F. Re, I. Cambianica, S. Sesana, E. Salvati, A. Cagnotto, M. Salmona, P.O. Couraud, S.M. Moghimi, M. Masserini and G. Sancini, *Journal of Biotechnology*, 2010, **156**, 341–346.

3 F. [Re](https://www.sciencedirect.com/science/article/pii/S1549963411001766?via%3Dihub#!), [I. Cambianica](https://www.sciencedirect.com/science/article/pii/S1549963411001766?via%3Dihub#!), [C. Zona](https://www.sciencedirect.com/science/article/pii/S1549963411001766?via%3Dihub#!), [S. Sesana](https://www.sciencedirect.com/science/article/pii/S1549963411001766?via%3Dihub#!), [M. Gregori](https://www.sciencedirect.com/science/article/pii/S1549963411001766?via%3Dihub#!), [R. Rigolio, B. La Ferla](https://www.sciencedirect.com/science/article/pii/S1549963411001766?via%3Dihub#!), [F. Nicotra](https://www.sciencedirect.com/science/article/pii/S1549963411001766?via%3Dihub#!), [G. Forloni](https://www.sciencedirect.com/science/article/pii/S1549963411001766?via%3Dihub#!), [A. Cagnotto](https://www.sciencedirect.com/science/article/pii/S1549963411001766?via%3Dihub#!), [M. Salmona](https://www.sciencedirect.com/science/article/pii/S1549963411001766?via%3Dihub#!), [M. Masserini](https://www.sciencedirect.com/science/article/pii/S1549963411001766?via%3Dihub#!) and [G. Sancini](https://www.sciencedirect.com/science/article/pii/S1549963411001766?via%3Dihub#!), [*Nanomedicine: Nanotechnology, Biology and Medicine*](https://www.sciencedirect.com/science/journal/15499634), 2011, [**7**,](https://www.sciencedirect.com/science/journal/15499634/7/5) 551-559.

4 L. Bana, S. Minniti, E. Salvati, S. Sesana, V. Zambelli, A. Cagnotto, A. Orlando, E. Cazzaniga, R. Zwart, W. Scheper, M. Masserini and F. Re, *Nanomedicine: Nanotechnology, Biology and Medicine*, 2014, **7**, 1583-1590.

5 R. Mare, D. Paolino, C. Celia, R. Molinaro, M. Fresta and D. Cosco, *Int J Pharm*, 2018, 552, 414-421.

6 J.C. Stewart, *Anal. Biochem*., 1980, **104**, 10–14.

7 S. Mancini, C. Balducci, E. Micotti, D. Tolomeo, G. Forloni, M. Masserini and F. Re, *Journal of Controlled Release*, 2017, **258**, 121-129.

8 M. Tamborini, E. Locatelli, M. Rasile, I. Monaco, S. Rodighiero, I. Corradini, M.C. Franchini, L. Passoni and M. Matteoli, *ACS Nano*, 2016, **10**, 2509-2520.

9 Dal Magro R, Simonelli S, Cox A, Formicola B, Corti R, Cassina V, Nardo L, Mantegazza F, Salerno D, Grasso G, Deriu MA, Danani A, Calabresi L and Re F, *Front Neurosci*, 2019, **13**, 1-15.

10 R. Cecchelli, B. Dehouck, L. Descamps, L. Fenart, V.V. Buée-Scherrer and C. Duhem, *Adv Drug Deliv Rev*, 1999, **36**, 165-78.

11 Y.T. Chen, W.C. Chao, H.T. Kuo, J.Y. Shen, I.H. Chen, H.C. Yang, J.S. Wang, J.F. Lu, R.P. Cheng and P.T. Chou, *Biochem Biophys Rep*, 2016, **7**, 113-118.

12 J. Huang, S. Rauscher, G. Nawrocki, T. Ran, M. Feig, B.L. De Groot, H. Grubmüller and A.D. MacKerell, *Nat. Methods*, 2016, **14**, 71–73.

13 W.L. Jorgensen, J. Chandrasekhar, J.D. Madura, R.W. Impey and M. Klein, *J. Chem. Phys*., 1983, **79**, 926–935.

14 M.J. Abraham, T. Murtola, R. Schulz, S. Páll, J.C. Smith, B. Hess and E. Lindahl, *SoftwareX*, 2015, **1–2**, 19–25.

15 R. Fletcher and M.J.D. Powell, *Comput. J.*, 1963, **6**, 163–168.

16 G. Bussi, D. Donadio and M. Parrinello, *J. Chem. Phys.,* 2007, **126**, 014101.

17 H.J.C. Berendsen, J.P.M. Postma, W.F. Van Gunsteren, A. DiNola and J.R. Haak, *J. Chem. Phys*., 1984, **81**, 3684–3690.

18 D.J. Evans and B.L. Holian, *J. Chem. Phys.*, 1985, **83**, 4069–4074.

19 B. Hess, H. Bekker, H.J.C. Berendsen and J.G.E.M. Fraaije, *J. Comput. Chem.,* 1997, **18**, 1463–1472.

20 T. Darden, D. York and L. Pedersen, *J. Chem. Phys*., 1993, **98**, 10089.

21 W. Humphrey, A. Dalke and K. Schulten, *J. Mol. Graph*., 1996, **14**, 33–38, 27–28.
